# Tumor Volumes and Prognosis in Laryngeal Cancer

**DOI:** 10.3390/cancers7040888

**Published:** 2015-11-10

**Authors:** Mohamad R. Issa, Stuart E. Samuels, Emily Bellile, Firas L. Shalabi, Avraham Eisbruch, Gregory Wolf

**Affiliations:** 1 Department of Otolaryngology/Head and Neck Surgery, The University of Michigan Health System, 1903 Taubman Bldg, 1500 East Medical Center Drive, Ann Arbor, MI 48109, USA; moeissa@med.umich.edu (M.R.I.); fshalabi@umich.edu (F.L.S.); 2 Department of Radiation Oncology, The University of Michigan, 1500 E Medical Center Drive, Ann Arbor, MI 48109, USA; samuelss@med.umich.edu (S.E.S.); eisbruch@med.umich.edu (A.E.); 3 Department of Biostatistics, The School of Public Health, University of Michigan, Ann Arbor, MI 48109, USA; lighte@med.umich.edu

**Keywords:** tumor volume, laryngeal cancer, prognosis, radiation therapy

## Abstract

Tumor staging systems for laryngeal cancer (LC) have been developed to assist in estimating prognosis after treatment and comparing treatment results across institutions. While the laryngeal TNM system has been shown to have prognostic information, varying cure rates in the literature have suggested concern about the accuracy and effectiveness of the T-classification in particular. To test the hypothesis that tumor volumes are more useful than T classification, we conducted a retrospective review of 78 patients with laryngeal cancer treated with radiation therapy at our institution. Using multivariable analysis, we demonstrate the significant prognostic value of anatomic volumes in patients with previously untreated laryngeal cancer. In this cohort, primary tumor volume (GTV_P_), composite nodal volumes (GTV_N_) and composite total volume (GTV_P_ + GTV_N_ = GTV_C_) had prognostic value in both univariate and multivariate cox model analysis. Interestingly, when anatomic volumes were measured from CT scans after a single cycle of induction chemotherapy, all significant prognosticating value for measured anatomic volumes was lost. Given the literature findings and the results of this study, the authors advocate the use of tumor anatomic volumes calculated from pretreatment scans to supplement the TNM staging system in subjects with untreated laryngeal cancer. The study found that tumor volume assessment after induction chemotherapy is not of prognostic significance.

## 1. Introduction

In the United States, head and neck cancer accounts for 3% of malignancies, with almost 60,000 Americans developing head and neck cancer annually and 12,000 dying from the disease [1]. Laryngeal cancer (LC) makes up about 14,000 cases in the U.S. annually. Laryngeal cancer is more likely to affect males, with an approximate 50% higher incidence in African American men [2]. Symptoms of LC can include hoarseness, dysphagia, otalgia and hemoptysis and, although hoarseness can be an early symptom, more than half of patients present with advanced disease with an overall five year survival rate around 50% (SEER data). Tumor staging systems have been developed to assist in estimating prognosis after treatment and compare treatment results across institutions [3]. There are significant variations in tumor staging characteristics across various tumor sites and across anatomic regions within any major tumor site. Many questions have been raised regarding which clinical features of a larynx cancer are of greatest importance for the TNM staging systems ability to estimate prognostic outcomes after treatment for LC. Effort has been made to utilize tumor volume measurements to predict response to treatment [4]. Tumor volumes could potentially supplement TNM staging information to select the optimal therapy for each individual patient. Here we present a brief history along with modern treatment paradigms for LC, followed by a review of the literature regarding the prognostic limitation of the TNM staging system and prognostic value of various tumor volumes following definitive treatment. Additionally, we will present our institutional experience regarding the prognostic value of tumor volume measurements.

## 2. Treatment

### 2.1. Early LC

Approximately 30% to 40% of patients with head and neck squamous cell carcinomas present with small volume, Stage I or II (early stage) disease. Glottic cancer accounts for approximately two-thirds of all LCs [5,6]. Supraglottic cancers, which constitute about one-third of LCs, are generally larger volume and more aggressive than glottic cancers [6]. Trials in recent decades have demonstrated a shift towards definitive radiation therapy (RT) or combined chemoradiation and endoscopic laryngeal preservation surgery over open surgery due to voice preservation. The results from research studies show similar survival rates with radiotherapy compared to primary surgery [7,8,9,10,11,12,13,14]. Local control, laryngeal preservation, and survival rates are similar with RT, transoral laser surgery, and open partial laryngectomy (open vertical hemilaryngectomy). Most patients diagnosed with early LC in Western countries are now treated with either RT or endolaryngeal surgery. The five-year cause-specific and overall survival rates are approximately 95% and 80% for patients with early glottic cancers [9]. Five-year disease-specific survival rates with both RT and laryngeal preservation surgery approaches are above 90% for stage I disease and around 80% for stage II tumors based on observational studies [15]. RT is often preferred because functional outcomes contributing to quality of life, especially voice quality, are better preserved, particularly for T2 cancers. A small randomized trial compared RT with laser surgery in 60 men with stage I disease (T1aN0M0) found that patients treated with RT (*n* = 32) reported less hoarseness-related inconveniences two years after treatment than those treated with laser surgery (*n* = 28). Voice quality was assessed at baseline and 6 and 24 months after treatment, with the specific outcome measures being the GRABS scale (subjective assessment of voice quality), videolaryngostroboscopic findings, and the patients' self-rated disease impact on activities of daily living [16]. Many factors, including treatment, invasiveness, quality of life and voice quality, are considered when selecting the best therapy for early laryngeal cancers.

### 2.2. Advanced LC

Locoregionally advanced (stage III/IV) squamous cell carcinoma of the larynx is associated with a high risk of both local recurrence and distant metastases [17]. Historically, total laryngectomy was the standard for treating locally-advanced LCs, although many patients and clinicians opted for definitive radiation with surgery held in reserve for salvage in the hope of avoiding total laryngectomy. Early reports of this treatment approach demonstrated potential survival benefit with the use of chemotherapy and radiation [14,18,19]. The landmark VA study was the first randomized, prospective study assessing organ preservation therapy with induction chemotherapy followed by radiation therapy, *versus* definitive surgery. The results demonstrated similar 2-year survival rates between the two groups (68%), with a high rate (64%) of organ preservation in the nonsurgical group [20]. Additional studies have corroborated the VA study results, including the European cooperative trial EROTC 24781 for patients with epilaryngeal and hypopharyngeal cancer [21] and the Radiation Oncology Group (RTOG) 91-11 intergroup study comparing induction chemotherapy followed by radiation, concurrent chemotherapy and radiation, and radiation therapy alone [22]. The RTOG 91-11 phase III study demonstrated the superiority of concomitant chemoradiation for laryngeal preservation, finding similar 3 and 10 year survival rates among those who received concomitant chemoradiation or induction chemotherapy, but significantly higher organ preservation rates in those who received concomitant chemoradiation [22]. These studies provided the foundation on which current treatment recommendations of advanced LCs are based.

Other studies have since established the role of concomitant chemoradiation to treat laryngeal cancer. The Meta-Analysis of Chemotherapy on Head and Neck Cancer (MACH-NC) pooled individual patient data from 93 trials and 16,485 patients with resectable or unresectable oral cavity, oropharyngeal, hypopharyngeal and LCs [23]. In all trials, patients were randomly assigned to definitive locoregional therapy alone (surgery and/or RT) or definitive locoregional therapy in combination with chemotherapy (induction, concurrent, or adjuvant). The analysis demonstrated that for LC concurrent chemoradiotherapy is more effective compared to induction chemotherapy followed by radiation (hazard ratio [HR] for death 0.87, *p* < 0.05) [24,25]. MACH-NC demonstrated a significant decrease in the risk of death compared with definitive local therapy alone when using concurrent chemotherapy (hazard ratio [HR] 0.81, 95% CI 0.78–0.86). These results have prompted the widespread use of this technique in patients with good baseline performance status. The MACH-NC meta-analysis found a greater benefit for platinum-based regimens as compared with other chemotherapy regimens for concurrent chemoradiation. The current recommendations are to use platinum-based chemotherapy regimens every three weeks, although weekly platinum based regimens may be just as effective and have less toxicity [26].

When combined with post-op RT or chemoRT, recent advances in surgical techniques allow for laryngeal preservation, often via minimally invasive surgery, in carefully selected patients. These include patients with T3 supraglottic cancer with minimal or moderate pre-epiglottic invasion. This approach has been found to have good outcomes, with the literature reporting 5 year local control rates of 69%–76% and an overall survival rate of 37% [12,27,28]. However, these surgical approaches have not been directly compared to chemoradiotherapy [29]. Further studies are needed to understand the role of these surgical techniques in the management of advanced LC.

Controversy exists regarding the optimal treatment for T4 tumors with limited cartilage invasion. The VA study initially demonstrated higher rates of salvage surgery in T4 tumors (56% *vs.* 29% other T-class tumors; *p* < 0.05). The finding of worse outcomes with T4 cancers prompted RTOG 91-11 to exclude T4 cancers from their study. The University of Michigan (U of M) 95-20 phase II clinical trial in patients with stage III/IV laryngeal cancer attempted to address this issue. The authors found comparable larynx preservation rates and survival outcomes in T4 tumors with cartilage invasion as T3 tumors in a cohort stratified to organ preservation techniques by a response to one cycle of induction chemotherapy [30]. Further studies are needed to fully understand the clinical variables in T4 laryngeal cancers that would warrant surgical therapy over organ preservation techniques.

The role of radical surgery in the treatment of LC has seen many changes. The findings of equivalent survival with superior organ-function preservation rates with organ preservation techniques relative to radical surgeries have resulted in most centers utilizing concurrent chemoradiation therapy, or, in highly selected patients, open organ preservation surgery, reserving salvage total laryngectomy for recurrences [26,31]. Surgical management still has a role in the treatment of T4 cancer as well as in patients who are not candidates for organ preservation treatment, including elderly patients, patients with resectable tumors that have destroyed vocal cords, those with extensive cartilage destruction from the primary tumor and for patients requiring surgical salvage for recurrent cancer [26].

## 3. LC and TNM Stage

The Tumor Node Metastasis (TNM) classification was introduced in 1944 by Pierre Denoix [32]. In 1987, the main LC staging systems, the International Union Against Cancer (UICC) and the American Joint Committee on Cancer (AJCC) staging systems, were unified. Staging systems were developed to reflect difference in outcomes based on clinical tumor characteristics and often incorporate tumor size. These staging systems were created to assist with prognosis, treatment selection, and research efforts, including comparisons of outcomes and driving clinical trial methodology [33]. The prognosis of laryngeal cancer depends largely on the stage of presentation, with the single most important factor being the presence of neck node metastases, which reduces long-term survival by 50% [34]. The exact form of treatment is usually based on a combination of tumor stage, patient desire, and institutional preference [35]. Clinical characteristics that would reliably predict the success of definitive treatment would be of considerable benefit in selecting patients for organ preserving strategies of radiation alone or chemoradiation.

Researchers have demonstrated the prognostic value of the TNM staging system in laryngeal cancer. In a retrospective review of 519 early glottis cancer patients, Mendenhall *et al.* demonstrated the importance of the TNM classification system by showing in a multivariate analysis that T stage significantly influences the local control rate (*p* < 0.05) [36]. Overall treatment time and histologic differentiation were also found to have prognostic value in this study. Murakami *et al*. (2004) showed the importance of (UICC) TNM staging in driving therapy in a retrospective review of 132 patients with T1-T3 glottic cancer by demonstrating that T-stage was a significant independent predictor for death, validating the use of more aggressive therapy in subjects with higher T stage [37]. Burke *et al.* demonstrated that T-stage was significant in local control (*p* < 0.05) in a retrospective review of 102 early glottis cancer patients, underlining the treatment-related implications in T2 tumors with inferior tumor control [38].

The laryngeal staging system has also been used to prognosticate quality of life. Ringash *et al.* demonstrated that patients with more advanced T-categories had lower QOL (quality of life) scores, assessed using the Functional Assessment of Cancer Therapy Head and Neck (FACT-H&N) questionnaire [39]. In a retrospective review of 164 supraglottic LCs comparing outcomes of surgery *versus* radiotherapy treatment, Santos *et al.* demonstrated in a multivariate analysis that only initial treatment, T and N stage had prognostic value for survival [40]. Further studies have validated the adverse influence of increasing T stage and overall stage on local control and severe complications [41,42].

While the Laryngeal TNM system has been shown to have prognostic information, some have expressed concerns about the weakness of the T-classification, as the cure rates reported in the literature vary [43]. While many staging systems factor in tumor volume, the current staging system for laryngeal carcinoma is only loosely related to tumor bulk [44]. For example, staging systems for tumors in the oropharynx and oral cavity factor in tumor diameter, while in the larynx, tumor extension is the key criteria [45]. Several studies have demonstrated that the current TNM staging systems fails to define the true three-dimensional bulk of head and neck tumors [46,47]. Pameijer *et al.* assessed the variability of tumor volumes in 12 subjects with T3 LCs, finding a striking variation in tumor volumes with variations exceeding 100% within the same T class (range 1.6 cm^3^–17 cm^3^; *p* < 0.05) [32]. This is concerning given the use of TNM in driving treatment options. A model of cell population kinetics during radiotherapy revealed a 10%–20% difference in the local control rate for tumors with similar diameters but different width (3 *vs.* 5 cm) [48]. Smaller tumors, which have fewer clonogenic and radioresistant cells may require different treatment relative to larger tumors of the same T stage.

The limitations of the laryngeal staging systems most often used (UICC and AJCC) have been demonstrated by Groome *et al.*, who compared seven systems with respect to prognosis for 861 larynx cancer patients from Canada and 642 from Norway [49]. They found UICC/AJCC-5 stage groupings did not perform well compared to other more empirical systems, and advocated the redefinition of LC stages in UICC/AJCC staging systems utilizing more empirical information. Researchers have demonstrated other limitations of the TNM staging system, identifying ways to further stratify patients and improve the TNM prognostic value. In addition to revealing the importance of T stage on a multivariate analysis, Mendenhall *et al.* highlighted limitations of the TNM classification system by showing in the retrospective review that vocal cord mobility can be used to further subdivide T2 cancers [36]. Mendenhall demonstrated that T2 cancers with intact vocal cord mobility had better outcomes in response to radiation treatment relative to those with T2 cancers with impaired vocal cord mobility (5 year local control 80% *vs.* 72%), suggesting that these cancers might benefit from more aggressive therapy. Burke *et al.* utilized the staging criteria suggested by Mendenhall *et al.* and he demonstrated findings consistent with Mendenhall *et al.*, showing that impaired vocal cord mobility leads to worse outcomes (5-year local control rate 94% *vs.* 83%; *p* < 0.05).

TNM staging is helpful because it provides prognostic information that guides treatment for cohorts of LC, but is limited by its inability to predict success on an individual basis. Other complementary methods have been studies to improve the prognosticating of outcomes, including TV. A quantitative analysis of imaging findings offers complementary information regarding the prognosis of head and neck tumors [4].

## 4. Imaging

Imaging methods, which are capable of characterizing the local extension and metastasis are key tools in the staging of LCs [4]. Although still somewhat controversial in early laryngeal cancers, routine pretreatment imaging has been shown to improve the accuracy of clinical staging and has become complementary to the physical examination for evaluation of most laryngeal tumors at many institutions [50,51,52]. Clinical examinations, including endoscopy, provide the most accurate evaluation of superficial cancer spread. Radiological examination and direct laryngoscopy under anesthesia provide the most accurate assessment of deep, local and regional spread of the neoplasm [53]. If radiological imaging is being obtained to help appropriately stage a tumor, objective measures such as TV are inexpensive tools that could potentially supplement prognostic information provided by the TNM staging system to help determine the best treatment options for every individual patient.

Various forms of imaging technology are used in the evaluation of LC. Modern high resolution CT scans offer advantage over the MRI given their availability, speed and ability to eliminate artifacts of movements. MRI scans, which are hampered by respiratory motion, are slightly better at staging LCs given their advantage of superior contrast resolution, which could be helpful in assessing cartilage invasion, preepiglottic space invasion and also provide multiparametric imaging [53].

PET-CT scans utilize ^18^F-fluorodeoxyglucose to identify hypermetabolic activity that defines LC. The role of routine pretreatment PET-CT scan for staging of cancer is still controversial, but has established uses post therapy. Early studies reported that PET scans have altered treatment in a substantial number of patients [54,55]. Post therapy, PET-CT is helpful in assessing therapy response and tumor surveillance. PET-CT is also useful for diseases of unknown primary, equivocal nodal disease, and advanced cases where distant or nodal metastases are suspected [56,57]. A review by Messa *et al.* of the role of PET and CT in radiotherapy indicates that the accuracy of staging head and neck cancers, particularly N and M staging, has a higher accuracy when analyzed by PET, compared to CT and MRI. The reviewers also reference studies that demonstrate that node positive patients with a negative PET study 3–4 months after treatment do not require subsequent neck dissection and can be safely observed [58]. Further studies are needed to assess the utility of this technology in the treatment of LC but it is likely that functional assessments combined with anatomic tumor measurements will provide useful prognostic information compared to clinical examinations alone.

One concern regarding tumor volume assessment is the reliability of these measurements. Mukheriji *et al.* demonstrated a high inter-observer reliability on tumor volumes (TV) measurements made on CT scans of supraglottic cancers utilizing eight experienced head and neck radiologists and radiation oncologists from different institutions [59]. Gordon *et al.* used an interactive computer program to extract tumor volumes from MRI data in patients with pharyngeal carcinoma, obtaining 202 volumetric measurements in 17 patients using one neuroradiologist. They demonstrated good intra-observer reliability, leading the authors to conclude that TV are reproducible [60]. Other authors have raised questions on the variability of tumor volume measurements. Hermans *et al.* conducted a study assessing intra-observer and inter-observer tumor volume measurement variability using CT scans to have 13 laryngeal tumors (including five supraglottic carcinomas) measured four times by five readers. He demonstrated a significant difference in measurements between individuals. The authors recommend that one, experienced observer should calculate TVs [61]. Overall, the literature points to the reliability of these measurements if performed by experienced head and neck imaging experts.

## 5. Tumor Volume

Gross primary tumor volume (GTV_P_), nodal volume (GTV_N_) and composite (GTV_P_ + GTV_N_ = GTV_T_) tumor volumes have been touted as potential independent prognosticating factors in LC. Below is a review of the literature for these volumes.

## 6. Gross Tumor Volume (GTV_P_)

Given that tumor clonogen number increases linearly with tumor volume, much effort has been made to study the gross tumor volume at the primary site (GTV_P_) to correlate with tumor treatment response [4]. In Pameijer’s at al. report, T3 LCs ranged from 1.7 to 17 cm^3^. This wide range of GTV_P_ is in contrast to the narrower GTV_P_ ranges within a T-class in oropharynx carcinoma, which is staged by maximum diameter [47]. A retrospective review of 76 patients with glottic T1N0 or T2N0 SCC revealed that the extent of the tumor is the most important predictor of RT outcome in early glottic carcinoma, suggesting a classification and prognosis based on actual size of the tumor, rather than conventional T-grouping [6]. GTV_P_ could be used to supplement the prognostic information provided by TNM to help identify patients who would benefit from more aggressive treatment. Bentzen *et al.* randomly sampled 113 patients with supraglottic LC treated with radiotherapy and found in a multivariate survival analysis that tumor size and lymph node status had prognostic importance [62]. The role of GTV_P_ to help select treatment options is highlighted by Lo *et al.*, who demonstrated that within T2 glottic cancers, larger tumors with GTV_P_ of about 5 cm^3^ (2–2.5 cm in diameter) need an extra 6.5 Gy to achieve similar 3-year LC as small tumors with 0.5 cm^3^ (~1 cm in diameter) [63]. With the wide spread use of imaging tests to appropriately stage patients with LC, GTV_P_ represent an inexpensive objective tool to help drive patient therapy options.

Studies have shown that there is a stronger association between GTV_P_ and local control than between T and N classification and local control [35,64,65]. Strongin *et al.* demonstrated, in a mixed cohort of hypopharynx, oropharynx and laryngeal stage III-IV SCC carcinoma, that the interval to progression correlated with primary tumor volume, and that subjects with GTV_P_ > 35 cm^3^ had worse outcomes relative to subjects with GTV_P_ < 35 cm^3^ (5 year OS 41 *vs.* 84%; *p* < 0.05). On the multivariate analysis, GTV_P_ was noted to have prognostic significance for recurrence and survival, while neither T-stage nor N stage were significant factors for either outcome measure [66]. Studer *et al.* demonstrated in a cohort of patients with head and neck cancer treated with IMRT that there is a stronger association between primary tumor volume and local control than the association between T-stage and local control [67].

GTV_P_ is associated with other known prognostic indicators, strengthening the argument to utilize GTV_P_ to augment TNM. Studies have demonstrated an association between GTV_P_ and nodal status. Patients presenting with nodal metastasis have significantly higher GTV_P_ relative to those without nodal involvement; Rutkowski *et al.* demonstrated that patients with nodal metastases had a median GTV_P_ of 7.3 cm^3^, relative to a GTV_P_ of 1.5 cm^3^ in those without nodal metastases (*p* < 0.05) [63]. Others have demonstrated the prognostic role of GTV_P_ in assessing nodal control, with Studer *et al.* finding worse two year outcome with increasing GTV_P_ (2 year nodal control for 1–15 cm^3^ , >15–70 cm^3^ and >70 cm^3^ was 95%, 90% and 75%, respectively; *p* < 0.05) [68]. Other studies have demonstrated that larger laryngeal tumor volumes are associated with pathologic evidence of thyroid cartilage penetration. Kats *et al.* demonstrated in 49 patient who underwent a total laryngectomy had increasing rates of thyroid cartilage penetration with increasing tumor size, demonstrating significantly different rates for different GTV_P_ group sizes (<25 cm^3^, 25–50 cm^3^, >50 cm^3^; 23%, 17%, and 78%, respectively) [69]. They found that when patients were divided by laryngeal subsite, only supraglottic tumors retained statistically significant association between volume subgroup and thyroid cartilage penetration (*p* = 0.04). Others have found an association with GTV_P_ and histological grade.

The prognostic significance of tumor volume has also been demonstrated with multiple treatment regimens. Because of the radiobiologic rationale linking tumor size to effectiveness of therapy, much of the research on GTV_P_ and outcomes has been done on LC cohorts that have been treated with definitive radiation. Gilbert *et al.* demonstrated in a group of 37 T2-T4 LC patients that tumor volume is an important prognostic variable for disease-free interval in T2-T4 laryngeal carcinomas treated with radiotherapy [55]. Others have demonstrated the role of GTV_P_ in prognosticating outcomes in surgically treated LC. Kazmi *et al.* assessed GTV_P_’s association with surgical outcomes, finding that LCs with volumes greater than 46 cm^3^ were associated with poor prognosis [70]. Mukherji *et al.* further demonstrated the relevance of GTV_P_ measurements in prognosticating outcomes in surgically treated supraglottic tumors, demonstrating a 97% *vs.* 50% local recurrence rates utilizing 16cc as a cutoff [35]. Other researchers have demonstrated the relationship between GTV_P_ and outcomes in LCs treated with multiple modalities. Strongin *et al.* demonstrated in a cohort of 19 laryngeal cancer patients undergoing definitive chemoradiation on multivariate analysis that the primary tumor volume was the best predicator of recurrence and survival, findings that T and N stage were both non-significant factors [66].

The literature specifically on glottic cancer consists of small, retrospective studies that mostly demonstrate a prognostic role for anatomic volumes in predicting therapy response. In a retrospective review of a cohort of 115 T2 glottic patients treated with definite radiotherapy, Rutkowski *et al.* observed that a six-fold increase in initial GTV_P_ (on average from 0.5 to 3 cm^3^) translated into a decrease in 3-year local control rate of about 30% (from 89% to 57%) [71]. The literature points to significant outcome differences for glottis cancers with different sizes, with 3.5 cm^3^ found to be a key cutoff point. Lee *et al.* demonstrated significant differences in local control rates for T3 glottic tumors based on pretreatment CT tumor volume and analysis of the lesion spread pattern (GTV_P_ < 3.5 *vs.* >3.5 cm^3^, 92 *vs.* 33%, respectively; *p* < 0.05) [72]. Pameijer *et al.* demonstrated in 42 patients with T3 glottic carcinoma that tumors ≤3.5 mL had better local control relative to tumors >3.5 mL (85% *vs.* 25%) [32].

Similar to research in glottic cancers, several small, retrospective studies have been conducted in attempts to address potential prognostic role that GTV_P_ could play in determining response to treatment for supraglottic cancers. Several studies have identified a GTV_P_ cutoff of roughly 6 cm^3^ to guide risk stratification. Freeman *et al.* found in a sample of 31 supraglottic T1-T3 cancer patients treated with definitive radiation an independent effect of tumor size on local control, with tumors <6 cm^3^ having better rates relative to tumors >6 cm^3^ (83% *vs.* 46%) [73]. Mancuso *et al.* demonstrated in a study of 63 supraglottic with T2-T4 supraglottic cancer cancer patients that tumor volume (>6 cm^3^ *vs.* <6 cm^3^) was a prognostic factor for outcome response to radiation therapy (local control 89% *vs.* 40%). The same study found that GTV_P_’s were associated with two year local control rates, with GTV_P_ <6 cm^3^ demonstrating a higher rate of local control relative to tumors >6 cm^3^ (89% *vs.* 52%, respectively) [74]. Moreover, the likelihood of maintaining laryngeal function also varied with GTV_P_ (<6 cm^3^ *vs.* >6 cm^3^; 89% *vs.* 40%, respectively; *p* < 0.05) [74]. Others have found cutoffs close to these values, including Kraas *et al.* who analyzed 28 supraglottic patients’ GTV_P_ calculated from pretreatment CTs revealing that >8 cm^3^ tumor volume was indicative of worse two year local control (20%) *versus* tumors less than 8 cm^3^ (70%) [75].

While most studies demonstrate a relationship between GTV_P_ and therapy outcomes, some authors found an absence of this relationship. Mukherji *et al.* demonstrated in a group of 28 T2 glottic patients that there was no relationship between tumor volume and outcome. Most of the studies reviewed only utilized one experienced head and neck radiologist and Mukherji *et al.* used two head and neck radiologists to review the 28 CT scans retrospectively [76]. The small sample size and use of two radiologists could have limited their ability to identify a relationship between these GTV_P_ and outcome. Janssens *et al.* demonstrated in the prospective phase 3 ARCON trial studying the addition of carbon breathing and nicotinamide to accelerated radiotherapy that no correlation was found between both primary tumor volume and total nodal volume with local control [67]. Janssens utilized new techniques, including accelerated radiotherapy, which is proven to have superior local control secondary to reduction in overall treatment time. The use of these newer techniques could have resulted in a loss of GTV_P_’s prognostic value found in other studies.

## 7. Composite Nodal Tumor Volume (GTV_N_)

The literature on composite nodal tumor volume is very limited. Most studies assessing GTV_N_ analyze this variable as a sub-analysis along with GTV_P_ and GTV_C_, rarely looking at the impact of GTV_N_ alone. These studies are small, retrospective analysis but results have consistently demonstrated the potential utility of GTV_N_ as a prognostic indicator to further supplement TNM staging system to best guide cancer therapy options. Currently, the maximum diameter of regional lymph nodes is utilized in the LC TNM staging system to identify patients who would benefit from further therapy, including neck dissection or chemoradiotherapy [3]. Given that this measurement does not represent the total nodal burden, authors have attempted to assess the utility of GTV_N_ as a prognostic variable in assessing therapy options for laryngeal cancers.

Taylor *et al.* demonstrated in a cohort of 140 head and neck cancer patients, 23 of which had laryngeal cancer, that increasing maximum lymph node diameter resulted in decreased local control. They reported the potential use of GTV_N_ as a prognostic variable by estimating that the radiotherapy dose required to achieve equivalent outcomes of a doubling in node volume is 4.2 Gy [77]. The different radiation dose required to achieve similar outcomes for nodes that are in the same TNM stage supports the use of GTV_N_ as a variable to help determine which patient might require more aggressive treatment, including increased radiation dose or surgery. Vergeer *et al.* demonstrated in a cohort of 79 head and neck SCC, 22 of which had laryngeal cancer, treated with either primary irradiation or chemoradiation, that total nodal volume and the use of chemotherapy were significant prognostic factors on multivariate analysis. These improved outcomes with smaller composite nodal tumor volumes was seen in both the cancer group treated with radiation alone (GTV_N_ <14 cm^3^ *vs.* >14 cm^3^; 2 year locoregional control 96% *vs.* 57%, respectively; *p* < 0.05), and the group treated with radiation and chemotherapy (GTV_N_ <14 cm^3^ *vs.* >14 cm^3^; 2 year locoregional control 91% *vs.* 64%, respectively; *p* < 0.05) [78]. In a cohort of 270 T2-T4 LC patients treated with accelerated radiotherapy with or without carbogen breathing and nicotinamide, Janssens *et al.* demonstrated a strong correlation between total nodal volume and N stage, but he did not find an association between primary tumor volume or total nodal volume with recurrence rate when analyzing the entire cohort. In a sub-analysis of each lymph node of the specific treatment arms, Janssens *et al.* demonstrated an inferior 5 year nodal control rate for larger nodal volumes with a cut-off of 3.5 cm^3^ (AR alone 79% *vs.* 54%; AR with carbogen/nicotinamide 98% *vs.*80%; *p* < 0.05) [67].

Other studies have shown that nodal volume is not a significant predictor of outcome in laryngeal cancer. Grabenbauer demonstrated in a group of 78 patients with stage III and IV locally advanced carcinoma of larynx or pharynx treated with radiation alone or with chemoradiation that on multivariate analysis, TV was a significant predictor of progression free survival but neither the nodal disease volume nor the total sum of all disease (primary and nodal) were statistically significant prognostic factors [79]. This could be due to the fact that this cohort received two different treatment modalities to treat this advanced cancer, one of which (radiation alone) is rarely utilized currently as a monotherapy. Doweck conducted a retrospective review of 64 patients with stage III–IV disease treated with chemoradiotherapy, finding that nodal volume did not have a statically significant prognostic role on logistic regression. Although the relationship did not reach statistical significance, Doweck did demonstrate a trend of worse outcomes in patients with larger composite nodal values [78].

The research available on GTV_N_ often represents a heterogeneous mixture of cancer sites, radiosensitivity, and treatment strategies, making it difficult to assess the true utility of this measure in laryngeal cancer. Given these limitations, these small studies provide evidence that total nodal volume is a significant prognostic factor for regional control. Given the use of imaging in staging laryngeal cancer, this measurement is another low-cost variable that could help supplement TNM and provide better prognosticating information for each individual patient. Further research is needed to fully understand the role of GTV_N_ in prognosticating laryngeal cancer outcomes.

## 8. Composite Tumor Volume (Composite Nodal Plus Primary; GTV_C_)

Similar to the literature on nodal tumor volume, the literature on composite tumor volume is limited with most studies assessing GTV_P_ and GTV_N_ along with GTV_C_. The European Organization for Research and Treatment of Cancer (EORTC) randomized trial on three fractions per day and misonidazole in a cohort of 523 patients with advanced head and neck cancer, 78 of which had laryngeal cancer, demonstrated on univariate analysis that both tumor and nodal volumes significantly influenced locoregional control. The study found that only GTV_C_ demonstrated significance on the multivariate analysis assessing variable influences on locoregional control [80]. Others have demonstrated similar findings. Plataniotis *et al.* demonstrated in a cohort of 101 stage III or IV head and neck cancer subjects, 51 of which had laryngeal cancer, that on a univariate analysis the significant parameters for survival included GTV_P_, GTV_N_, and GTV_C_ [81], while only GTV_C_ maintained significance in the multivariate analysis. Analysis revealed a prognostic cutoff point of 22.8 cm^3^, with those having smaller tumors demonstrating better outcomes (<22.8 cm^3^ *vs.* >22.8 cm^3^, median survival 12.3 months *vs.* 45.3 months, *p* < 0.05). Rudat *et al.* assessed a cohort of 68 stage IV head and neck cancer patients, nine of which had laryngeal cancer, treated with radiochemotherapy (3-year OS rate of 35%) [8]. The pretreatment GTV_C_ was significantly associated with survival (*p* < 0.05), with individuals with larger tumors demonstrating worse outcomes (<112.3 mL *vs.* 112.3 mL, 3 year OS 49 *vs.* 19%, *p* < 0.05). Johnson *et al.* demonstrated in a group of 76 advanced head and neck SCC patients treated with concomitant boost accelerate super fractionated irradiation that GTV_C_ <35 cm^3^ had better outcomes relative to tumors >35 cm^3^ (3 year local control 92 *vs.* 34%, *p* < 0.05). GTV_C_ was a significant prognostic variable on both univariate and multivariate analysis. Although the prognosis cutoff point in the studies reviewed vary, much of this can be attributed to the different treatment modalities utilized in the various studies [46].

Other researchers have demonstrated the GTV_C_ does not have prognostic significance. As highlighted in the GTV_N_ section, Grabenbauer *et al.* demonstrated that the composite tumor volume did not have prognostic value in his study of 78 cancer patients. The absence of a relationship between GTV_C_ and outcomes could be secondary to the heterogeneous nature of both the cancer characteristics and treatment modalities. Further, the implications of Grabenbauer *et al.*’s findings are limited as one of the treatment modalities utilized in the study is rarely used to treat advance laryngeal cancers alone [79].

These studies have many limitations, including heterogeneous cohorts and treatment modalities that limit the ability of researcher to fully assess the relationship between composite tumor volume and outcome in laryngeal cancer. Given these limitations, the literature does point to the potential role for GTV_C_ in supplementing the TNM staging system in laryngeal cancer to allow for the most accurate treatment stratification. Further studies are needed to be able to fully understand the role that GTV_C_ plays in prognosticating outcomes in laryngeal cancer patients.

### 8.1. The U of M Experience

The literature indicates that there is a prognostic role for anatomic tumor volumes in predicting treatment outcomes in patients with laryngeal cancers. Anatomic volume could be inexpensively, readily calculated with a high degree of reliability from staging scans or pre-treatment imaging. These measurements could supplement the TNM system to help identify patients that benefit from early, aggressive therapy. In order to assess and confirm the prior findings of associations between tumor volume and prognosis in patients with laryngeal cancer treated with definitive therapy, we undertook a retrospective study of 78 patients from our institution with laryngeal cancers to determine if GTV_P_, GTV_N_, or GTV_C_ are prognostic factors independent of TNM staging.

### 8.2. Methods

We performed an IRB approved retrospective chart review of patients with laryngeal cancer from the Specialized Program Of Research Excellence (SPORE) I and II databases. . Patients’ clinical and demographic information were recorded on registration into the SPORE database; clinical treatment and outcomes were monitored and recorded retrospectively by SPORE staff. This information was supplemented by information provided by further chart review.

**Figure 1:**
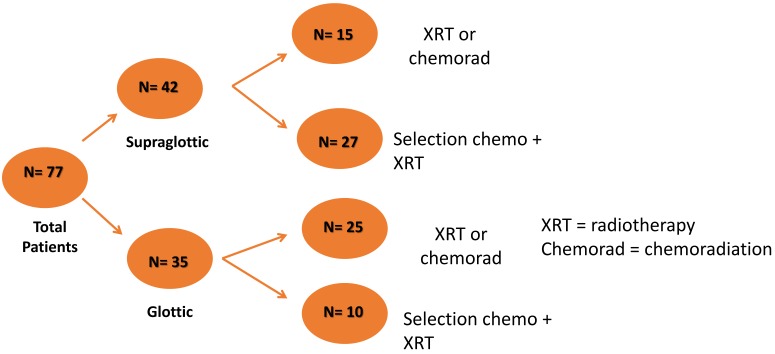
Schematic displays total cohort tumor primary site and initial treatment therapy.

Patients were enrolled between January 2003 and December 2012. Of the 169 patients enrolled, 82 were treated at the University of Michigan Radiation Oncology department and underwent pretreatment, contrast-enhanced planning CT scans that were available for review. Five patients had tumors with minimal response on induction chemotherapy; these patients were surgically treated and were excluded from the study. Thirty seven patients were treated with a single cycle of induction chemotherapy followed by concurrent chemoradiation (Figure 1). Forty patients did not receive induction therapy and were treated with either definitive radiation (*n* = 20) or concurrent chemoradiation (*n* = 20). These patients had similar baseline demographic and clinical characteristics along with similar survival outcome measures (Table 1). Kaplan Meier (KM) curves assessing survival revealed no difference in outcome (KM log rank statistic *p* > 0.05) between the two groups. The cohort treated with chemoradiation had a significantly higher recurrence rate (0.60% *vs.* 0.15%; *p* < 0.01); this cohort had a higher percentage of supraglottic tumors relative to the cohort treated with definitive radiation only (50% *vs.* 25%; *p* = 0.10). For the purpose of the analysis, the two cohorts were grouped together.

**Table 1:** Baseline demographic and clinical variables for the cohorts treated with definitive radiation or concurrent chemoradiation. Overall Survival Time (OST) and Recurrence Free Time (RFT) are reported.

		Concurrent Chemoradiation	Radiation Alone
Age		60.40(10.20)	64.2(11)
Gender	Male	16	19
	Female	4	1
Tobacco	Current (within 1 year)	10	11
	Former (quit >1 year), Never	10	9
Alcohol Use	Current (within 1 year)	7	7
	Former (quit >1 year), Never	13	13
Site	Glottic	10	15
	Supraglottic	10	5
Stage	Early (I/II)	6	5
	Advanced (III/IV)	14	15
Deaths		9	6
Recurrence		12	3
2 year OST		0.7	0.72
2 year RFT		0.43	0.84

### 8.3. Clinical Characteristics

Tumor staging was based on guidelines of the American Joint Committee on Cancer. We prospectively collected demographic and clinical data including age (years), sex (m/f), cancer site (glottis/supraglottis), stage (early (I/II) *vs.* Advanced (III, IV)), treatment (Definitive radiation/concurrent chemoradiation *vs.* selection chemotherapy followed by chemoradiation), comorbidities (Severe-none), smoking (Current/former,never), drinking (current/former,never), BMI and HPV status. Mean values and standard deviations (sd) are presented where appropriate. HPV status was determined by an ultra-sensitive method using real-time competitive polymerase chain reaction and matrix-assisted laser desorption/ionization-time of flight mass spectroscopy with separation of products on a matrix-loaded silicon chip array.

### 8.4. Treatment

Selection chemotherapy using a single cycle of neoadjuvant chemotherapy consisted of a platinum-based combination chemotherapeutic regimen. Specific drug combinations utilized included cisplatin and 5-fluorouracil (*n* = 22), cisplatin and docetaxel (*n* = 2), cisplatin and texotere (*n* = 1), carboplatin and 5-fluorouracil (*n* = 8), carboplatin and taxotere (*n* = 2), and carboplatin and docetaxel (*n* = 2). In this cohort, chemotherapy drugs used during concurrent chemoradiation included either cisplatin (*n* = 22), carboplatin (*n* = 23), carboplatin and 5-fluorouracil (*n* = 2), taxotere and xeloda (*n* = 2), erbitux (*n* = 2), cisplatin and paclitaxel (*n* = 1), cisplatin and 5-fluorouracil (*n* = 1), carboplatin and taxol (*n* = 3), carboplatin and etoposide (*n* = 1), or gemcitabine (*n* = 1).

Radiation treatment planning, doses and fractionation differed depending on the location and stage of each patient's disease. Patients with stage 1 or 2 glottic larynx cancers, were treated with lateral opposed fields covering the entire larynx to a total dose of 63 Gy or 65.25 Gy in 2.25 Gy fractions. Patients with stage 3 and 4 glottic or supraglottic larynx cancers, with a 0.5 cm expansion for clinical tumor volume (CTV) and an additional a 3 mm expansion for planning target volume (PTV) to account for setup uncertainties were treated to 70 Gy in 2 Gy fractions using intensity modulated radiation therapy (IMRT). High and low risk nodal regions were contoured as clinically indicated based on the extent and location of disease and were treated with 63 Gy in 1.8 Gy fractions, 59 Gy in 1.7 Gy fractions or 56 Gy in 1.6 Gy fractions depending on the clinical risk. Radiation doses to the pharyngeal constrictors (mean dose <50 Gy) and parotid glands (mean dose <24 Gy) were limited to decrease the risk of long term dysphagia and xerostomia.

### 8.5. Volume Measures

Primary tumor gross tumor volumes (GTV_P_) were contoured and calculated on contrast-enhanced treatment planning CT scans using the University of Michigan treatment planning software (UM plan). Planning CT scans were utilized given that diagnostic CT scans were not uniform and were not available for all subjects. Patients were immobilized during CT imaging using a 5-point thermoplastic mask. Tumor volumes (TVs) were drawn on 3 mm axial slices by radiation oncologists (SES, AE) as they appeared on imaging, without any expansions for microscopic disease or areas of clinical concern. Direct Laryngoscopy reports, PET/CT imaging, and MRI imaging, when available, were used as supplemental information and as anatomic guides but no images were registered to the CT scans. Separate volumes for the primary tumor (GTV_P_) and the lymph nodes (GTV_N_) were contoured and the total volume of all disease was calculated as a composite volume (GTV_P_ + GTV_N_ = GTV_C_). All volumes were calculated in cubic centimeters.

### 8.6. Statistical Methods

Nonparametric tests (Kruskal-Wallis and Wilcoxon-Mann-Whitney tests) were employed to assess for significant differences in volume measures between groups based on clinical characteristics (age, stage, tumor site, HPV status, BMI, HPV, smoking, drinking, comorbidities). Univariate survival analyses were performed utilizing Kaplan-Meier plots of volume measures and Cox proportional hazards models treating volume measures as continuous to determine hazard ratios for 1 cm^3^ increase in volume. Multivariable Cox Proportional Hazards models were utilized for volume measures in addition to historically important clinical variables.The dependent (outcome) variables were overall survival and recurrence free time. Overall survival time was defined from date of diagnosis by UM physician. Recurrence Free Time (RFT) time was defined as time from diagnosis to recurrence event or end of follow-up. End of follow-up is the last date where patient was reviewed for recurrence. Patients whose disease never cleared after treatment are considered recurrent with a recurrence time =1 day. All statistical analyses were performed using SAS 9.4 (Cary, NC, USA).

### 8.7. Results

Total number of patients in the cohort was 77, with a median age of 60 (range 33–85). 60 patients were male; 17 female (Table 2). Most study patients had a mild-moderate comorbidity (71%). HPV status was available on 26 patients, with four demonstrating positive results. The primary tumor site and stage breakdown of the total cohort was 18 early (stage I/II) glottic, 17 advanced (stage III/IV) glottic, three early supraglottic and 39 advanced supraglottic cancers. 26 patients were current tobacco users; 51 reported as former/never smokers. 26 patients used alcohol at the time of the study, with 48 reporting former or never use. Median anatomic volumes (range) for the total group were GTV_P_ 7.17 cm^3^ (0.3, 115.62), GTV_N_ 0 cm^3^ (0, 224.25), and GTV_C_ 8.33 cm^3^ (0.3, 332.5). Twenty seven patients either had tumor that persisted despite treatment or recurred (16 supraglottic, 11 glottic). The total group 2-year KM OS and RF was 0.75 and 0.68, respectively. Median follow-up for survival in the cohort was 18 months. Median follow up for recurrence was 13 months.

**Table 2:** Demographic and clinical characteristics of the total cohort, cohort treated with either definitive radiation or chemoradiation and the cohort treated with induction chemotherapy followed by chemoradiation. Overall Survival Time (OST) and Recurrence Free Time (RFT) are reported.

Characteristic		Total Cohort	Non-Induction Chemo Cohort	Induction Chemo Cohort
Age at Dx (Years) **^**		59.7(10.10)	62.3(10.70)	56.9(8.80)
Gender **^+^**	Male	60	35	25
	Female	17	5	12
Stage *	I/II	21	21	0
	III/IV	56	19	37
Comorbidities *	None	14	5	9
	Mild	35	16	19
	Moderate	20	11	9
	Severe	8	8	0
Tobacco use *	Current #	26	21	32
	Former/Never	51	19	5
Alcohol use *	Current #	26	14	12
	Former/Never	48	25	23
GTV_P_ **^**		7.17(0.3, 115.62),	6.94(0.3, 115.62)	10.19(2.6, 87.55)
GTV_N_ **^**		0(0, 224.25)	0(0, 224.25)	0(0, 28.23)
GTV_C_ **^**		8.33(0.3, 332.5)	6.94(0.3, 332.45)	10.19(2.6, 87.55)
2 year RFT **^**		0.68	0.63	0.70
2 year OST **^**		0.75	0.74	0.75

* Number of patients; ^ Median value (SD); ^+^ Mean value (SD); # Use in past 12 months.

40 patients were treated with either definitive radiation (*n* = 20) or concurrent chemoradiation (*n* = 20). The average age of these patients was 62 years (sd 10.7), with 35 males and 5 females. The site and stage breakdown for this group was 18 early glottic, seven advanced glottis, three early supraglottic and 12 advanced supraglottic cancer. Two year OST and RFT for the cohort treated with only definitive radiation was 0.74 and 0.63, respectively. Median anatomic volumes included GTV_P_ 6.94 cm^3^ (0.3, 115.62), GTV_N_ 0 cm^3^ (0, 224.25), and GTV_C_ 6.94 cm^3^ (0.3, 332.45).

Thirty seven patients were treated with induction chemotherapy followed by chemoradiation. The average age of this group was 57 years (sd 8.8) with 25M and 12 F. All patients who received chemotherapy had advanced cancer, with a primary site breakdown of 10 glottic and 27 supraglottic. Two year OST and RFT was 0.75 and 0.70, respectively. Median anatomic volumes included GTV_P_ 10.19 cm^3^ (2.6, 87.55), GTV_N_ 0 cm^3^ (0, 28.23), and GTV_C_ 10.19 cm^3^ (2.6, 87.55).

### 8.8. Tumor Volumes and Clinical Variables

**Figure 2:**
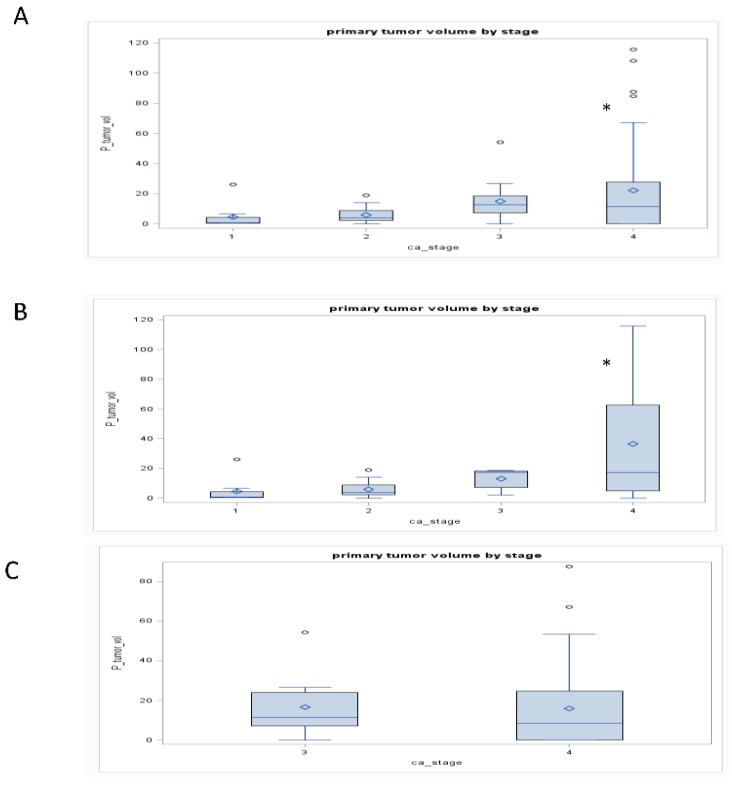
Boxplots demonstrate the relationship between GTVp and clinic stage for the total cohort (Panel A), cohort treated with chemoradiation or definitive radiation (Panel B), and the cohort treated with selection (induction) chemotherapy followed by chemoradiation (Panel C). * *p* < 0.05. The figure demonstrated an association between GTV_P_ and clinical stage for the total cohort that was maintained in the cohort treated with chemotherapy or definitive radiation but lost in the cohort treated first with selection (induction) chemotherapy.

**Figure 3:**
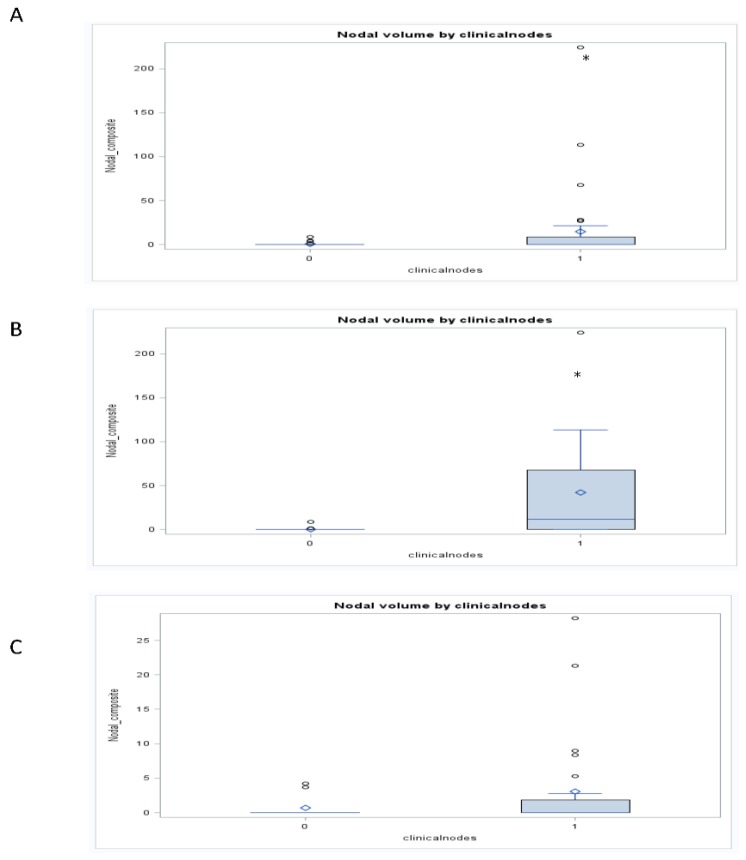
Boxplots demonstrate the relationship between GTV_N_ and clinic nodal status (1 *vs.* 0) for the total cohort (Panel A), cohort treated with chemoradiation or definitive radiation (Panel B), and the cohort treated with selection chemotherapy followed by chemoradiation (Panel C). * *p* < 0.05. The figure demonstrated an association between GTV_N_ and clinical nodal status for the total cohort that was maintained in the cohort treated with chemotherapy or definitive radiation but lost in the cohort treated first with induction chemotherapy.

For the entire cohort, tumor stage was associated with GTV_P_, GTV_N_, and GTV_C_ (*r* = 0.27, 0.15 and 0.24, respectively; *p* < 0.05; Figure 2) and clinical nodal status associated with GTV_N_ (*r* = 0.25; *p* < 0.05; Figure 3). When chemotherapy status was considered, the significant relationships between anatomic volumes with stage (GTV_P_ r = 0.31; GTV_N_ r = 0.31; GTV_C_ r = 0.42; *p* < 0.05) and clinical nodal status (GTV_P_ r = 0.0.52; GTV_N_ r = 0.47; GTV_C_ r = 0.55; *p* < 0.05) were maintained only in those who did not receive induction therapy. In this cohort, all three anatomic volumes were associated with clinical nodal status. In a subgroup analysis of glottic and supraglottic cancers treated with either definitive radiation or concurrent chemoradiation, both groups maintained a significant association between clinical nodal status and GTV_N_ (Glottic r = 0.45, supraglottic r = 0.4; *p* < 0.05). Only the glottic cohort demonstrated an association between tumor stage and anatomic volume, revealing significant associations with all three anatomic (GTV_P_ r = 0.52; GTV_N_ r = 0.38; GTV_C_ r = 0.43; *p* < 0.05). Subgroup analysis of those treated with selection chemotherapy followed by chemoradiation.

### 8.9. Tumor Volumes and Outcomes

**Figure 4:**
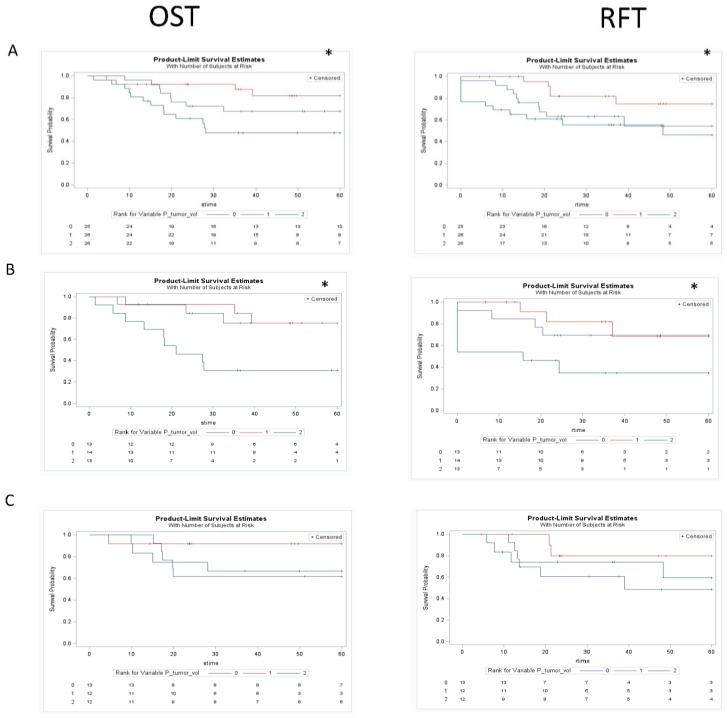
Kaplan Meier curves assessing the prognostic significance of GTV_p_ on both overall survival and recurrence free survival in the total cohort (row A), cohort treated with chemoradiation or definitive radiation (row B), and the cohort treated with selection chemotherapy followed by chemoradiaiton (row C). * *p* < 0.05. The figure demonstrated an association between GTV_P_ and clinical outcomes for the total cohort that was maintained in the cohort treated with chemotherapy or definitive radiation but lost in the cohort treated first with induction chemotherapy.

**Figure 5:**
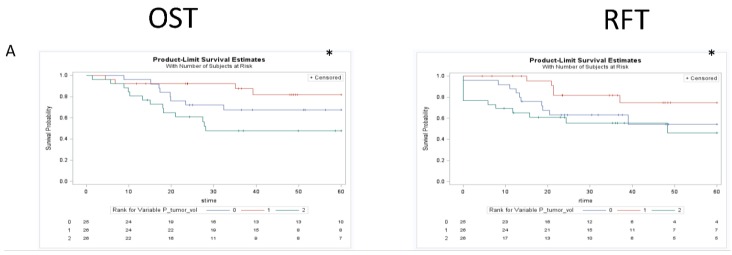

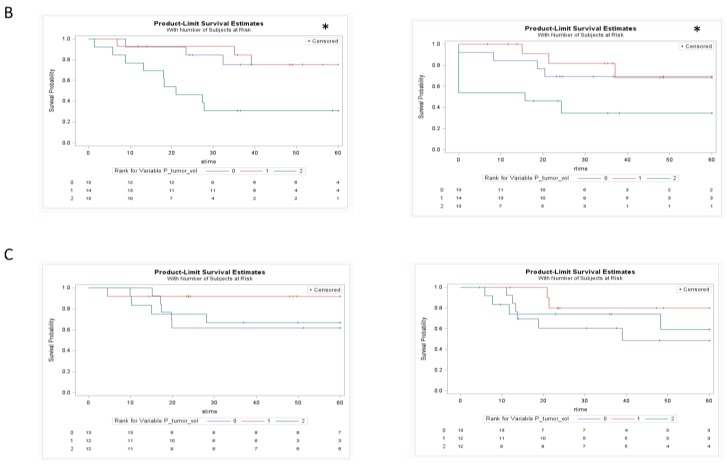
Kaplan Meier curves assessing the prognostic significance of GTV_N_ on both overall survival and recurrence free survival in the total cohort (row A), cohort treated with chemoradiation or definitive radiation (row B), and the cohort treated with induction chemotherapy followed by chemoradiation (row C). * *p* < 0.05. The figure demonstrated an association between GTV_N_ and clinical outcomes for the total cohort that was maintained in the cohort treated with chemotherapy or definitive radiation but lost in the cohort treated first with induction chemotherapy.

**Figure 6:**
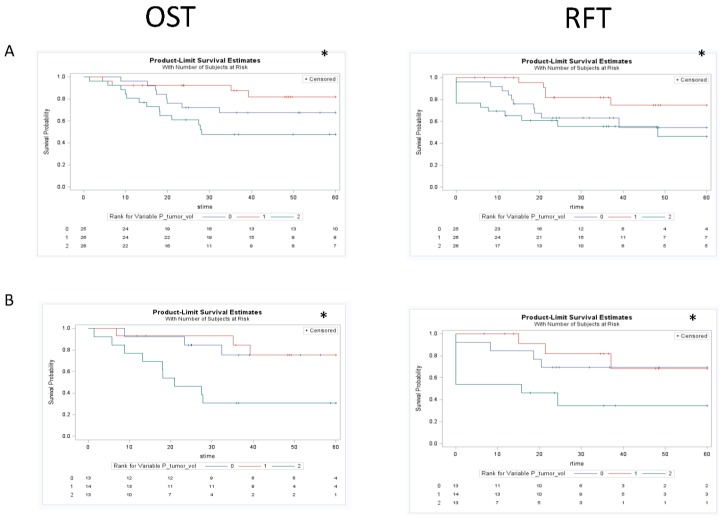

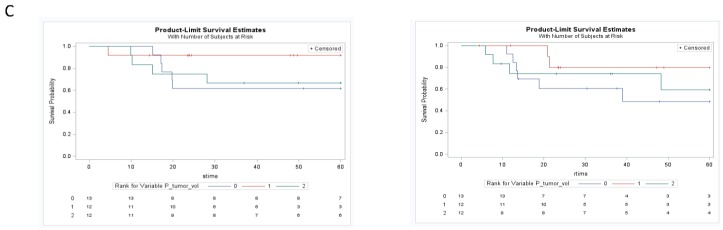
Kaplan Meier curves assessing the prognostic significance of GTV_C_ on both overall survival and recurrence free survival in the total cohort (row A), cohort treated with chemoradiation or definitive radiation (row B), and the cohort treated with selection chemotherapy followed by chemoradiaiton (row C). * *p* < 0.05. The figure demonstrated an association between GTV_C_ and clinical outcomes for the total cohort that was maintained in the cohort treated with chemotherapy or definitive radiation but lost in the cohort treated first with induction chemotherapy.

Univariate KM analysis for the total cohort utilizing three groups (based on tertiles) revealed significant differences in OST and RFT outcomes in the different groups for GTV_P_, GTV_N_, and GTV_C_ (Figure 4, Figure 5 and Figure 6). These findings were maintained in the group that did not receive induction chemotherapy, and were all lost in the group that had a response to induction chemotherapy. Subanalysis by primary site in those who did not receive any induction chemotherapy demonstrated maintenance of some of these findings, with both glottic (OST-GTV_P_, GTV_N_, GTV_C_; RFT-GTV_N_ ; *p* < 0.05) and supraglottic (OST-GTV_N_; RFT-GTV_N_, GTV_C_; *p* < 0.05) sites revealing outcome differences for different groups of anatomic volumes. The subanalysis by primary site in the group treated with induction chemotherapy followed by chemoradiation was only significant for different outcomes with GTV_N_ groups in the glottic site and GTV_C_ in the supraglottic site.

**Table 3:** Cox univariable and multivariable analysis are presented for the total cohort, cohort treated with chemoradiation or definitive radiation, and the cohort treated with induction chemotherapy. The volumes included in the multipvariavate analysis include GTV Primary (GTVp), Nodal volume (GTVn), Composite volume (GTVp + GTVn = GTVc). Other variables included in multivariate analysis included age, smoking status, stage and clinical node status. Clinical nodal status was used in the base model to assess whether volumes or clinical variables were better in prognosticating outcomes, as well as if the volumes were still useful after adjusting for the usual clinical variables. * *p* < 0.05. The table demonstrated significant relationships between anatomic tumor volumes and outcomes for the total cohort that was maintained in the cohort treated with chemoradiation or definitive radiation and loss of these relationships in the cohort treated with induction chemotherapy first.

		Tumor Volumes	n	Univariable HR (*p*-Value)	Multivariable HR (*p*-Value)
Total cohort	OST	GTVp	77	1.02(<0.01)	1.01(0.16)
		GTVn	77	1.02(<0.01)	1.02(<0.01)
		GTVc	77	1.01(<0.01)	1.01(<0.01)
	RFT	GTVp	77	1.01(0.02)	1.01(0.21)
		GTVn	77	1.01(<0.01)	1.01(<0.01)
		GTVc	77	1.01(<0.01)	1.01(<0.01)
Non-induction chemo cohort	OST	GTVp	40	1.02(<0.01)	1.02(0.04)
		GTVn	40	1.02(<0.01)	1.02(0.02)
		GTVc	40	1.02(<0.01)	1.01(0.01)
	RFT	GTVp	40	1.02(<0.01)	1.01(0.33)
		GTVn	40	1.01(<0.01)	1.01(0.2)
		GTVc	40	1.01(<0.01)	1.00(0.2)
Induction chemo cohort	OST	GTVp	37	1(0.90)	0.90(0.80)
		GTVn	37	1.05(0.35)	1(0.90)
		GTVc	37	1.00(0.70)	0.90(0.80)
	RFT	GTVp	37	0.90(0.90)	1(0.90)
		GTVn	37	0.70(0.30)	0.7(0.30)
		GTVc	37	0.90(0.70)	0.9(0.80)

Univariate Cox models with OST and RFT as outcome variables for the total cohort revealed significant HR for all three anatomic volume variables (OST GTV_P_ 1.02; GTV_N_ 1.02; GTV_C_ 1.02; *p* < 0.05). All cox uni- and multi-variable analysis results are found in Table 3. Multivariate Cox models in the entire cohort with outcome measures of OST and RFT adjusting for clinical variables (Age, Smoker, Stage, Clinical nodal status) along with the anatomic variables of interest revealed a significant role for GTV_N_, and GTV_C_, with GTV_P_ losing significance when adjusting for clinical variables (OST p = 0.16, RFT p = 0.21). The sub analysis of the cohort treated with either definitive radiation or chemo radiation maintained these findings with OST as the outcome of interest, along with finding that GTV_P_ was associated with OST outcomes in the multivariate analysis. The multivariate model using RFT as the outcome did not have any significant findings. In the subset of the cohort that received selection chemotherapy followed by chemoradiation, all relationships between anatomic volumes and outcomes were lost.

Models with traditional TNM clinical status *vs.* substituting volumes for T, N or both were created to directly assess the value of clinical markers *versus* volumes. The models indicate that using volume measures improved the model fit relative to the traditional T class and N class using both OST and RFT as outcomes measures.

## 9. Discussion

In our institutional retrospective series, anatomic tumor volumes demonstrated prognostic value in patients with previously untreated laryngeal cancers who did not receive induction chemotherapy. The widespread use of imaging technology to fully stage patients provides for the calculations of these volumes at little additional cost, assuming that a radiation oncologist or similar qualified professional can draw the volumes. However, measurements can be taken from treatment planning CTs and used in clinical algorithms to determine prognosis. Our study is consistent with other literature in demonstrating that anatomic tumor volumes are significant predictors of survival and recurrence in untreated subjects, including those not treated with induction therapy, with cancers originating at the glottis demonstrating stronger correlations with outcomes relative to supraglottic tumors. Similar to published reports, our patients with supraglottic tumors presented with larger median anatomic volumes (GTV_p_ 11.44 cm^3^ *vs.* 5.1 cm^3^), more advanced stage (92.8% advanced *vs.* 51% advanced) and larger number of recurrences (38.1% *vs.* 31.4%) relative to glottic cancers [65,82]. Tumors with lower anatomic volumes had improved outcomes and were associated with both a lower tumor stage and T-class. Interestingly, the relationships between anatomic tumor volume and outcomes for laryngeal cancer are lost when post-induction chemotherapy treatment scans are analyzed. This loss of relationship is presumably because the volumes were decreased after induction chemotherapy at the time of radiation treatment planning; measured anatomic volumes may have been related to outcomes if pre- chemotherapy CT scans were utilized for anatomic measurement. Our results suggest that tumor anatomic volumes calculated from pretreatment scans should be utilized to supplement the TNM system to identify patients who would benefit from more aggressive therapy and that significant responses to induction chemotherapy can eliminate the association of tumor volumes with prognosis.

Although this study confirms our hypothesis of the association of tumor volumes with outcomes, the study has several limitations. The study is small with subjects receiving heterogeneous treatments; thus the findings must be interpreted with caution. Further, our small retrospective analysis included primary information gathered from chart reviews. Given the heterogeneity of tumor stage and primary site of origin, and the small patient number, we had limited ability to assess the role of anatomic volumes in predicting outcome for all subgroups. The strength of the study is that the treatment provided to the cohort was consistent during the study period.

Our findings indicate that in the total cohort of laryngeal cancer patients, all anatomic volumes demonstrated prognostic significance on univariate analysis, with GTVN and GTVC maintaining their significant roles on the multivariable analysis. In addition, tumor volume data added prognostic value over just clinical staging parameters alone. Our results agree with findings of other authors reviewed in the introduction [65,75,82,83]. Rutkowski *et al.* demonstrated in a cohort of 160 patients (82 glottis, 78 epiglottis) that on both uni- and multivariate analysis, measured anatomical tumor volumes were significantly related to subject outcomes, including local control and overall survival.

Interestingly, in the group who received induction chemotherapy followed by radiation, all significant relationships were lost. Figure 4, Figure 5 and Figure 6 demonstrate KM univariate survival analysis and highlight these lost relationships for the cohort treated with induction chemotherapy. The response to treatment is influenced by tumor microenvironmental factors, such as tumor oxygenation, proliferation, intrinsic resistance, and acquired drug resistance [84]. Our results point to the heterogeneity of these factors resulting in variable tumor response to chemotherapy that could render anatomic measurements post treatment uninformative.

## 10. Future Directions

Advancements in imaging technologies have significant implications for the use of measured volumes in selecting patient treatments. These new technologies, often with better resolution than CT scans, can potentially provide more accurate tumor volumes, as well as employ tumor-specific biomarkers and other techniques to provide tumor measurements that may be useful in prognosticating laryngeal tumors. The use of PET/CT to calculate GTV_p_ is an exciting approach as the metabolic information provided by the PET scan along with the anatomic information from the CT could help more accurately measure the most meaningful PTV. Geetsa *et al.* demonstrated in a cohort of 18 patients with oropharynx or LC that GTV_p_ calculated from FDG-PET were significantly smaller than those based on pre-treatment CT [84]. A significant impact of the delineation of GTV_P_’s in laryngeal SCC translated into more normal tissue sparing after conformal radiotherapy planning. Others have demonstrated the ability of anti-EGFR (cetuximab) radiolabeled tracers with PET scans to monitor receptor expression in Head and Neck cancers [85]. Tumor Hypoxic index, given the established relationship between hypoxia and radiotherapy treatment resistance, is another factor that has been studied in patients with laryngeal cancers. Researcher have shown that PET scans can be utilized to reliably calculate tumors hypoxia index, with early findings demonstrating a correlation between hypoxic index with locoregional failure (LRF) and local recurrence (LC) [86,87,88].

Other interesting technologies include the use of diffusion weighted MRI scans. Diffusion-weighted (DW)-MRI is able to characterize tissue and generate image contrast based on differences in tissue water mobility [89]. The more water mobility measured, the less dense the tumor theoretically is. This work is supported by previous studies that show a correlation between signal intensity on diffusion weighted MRI and tumor cellularity in experimental models [90,91]. Vandecaveye *et al.* demonstrated the ability of DW-MRI scans to differentiate between persistent or recurrent Head and Neck Squamous Cell Carcinoma from post treatment, nontumor cells [92]. Future studies are needed to understand the role of these various, imaging advancements in prognosticating laryngeal cancer.

## 11. Conclusions

Even in our small clinical study, when pretreatment volumes are considered, correlation of anatomic TV with T class, clinical stage and outcomes (both OST and RFT) were significant. Tumor response after induction chemotherapy resulted in a loss of the relationship. Our small study must be interpreted with caution given the heterogonous treatment therapy received by subjects. In the context of the literature, our study supports the use of tumor volumes to supplement TNM staging. The use of pre-treatment CT scans to supplement the TNM staging system could help identify patients who would benefit from aggressive early therapy to help provide the best treatment options for all patients.
